# Evaluation of Open (Hasson's) and Closed (Veress) Technique of Intraperitoneal Access for Creation of Pneumoperitoneum in Laparoscopic Surgery

**DOI:** 10.7759/cureus.54770

**Published:** 2024-02-23

**Authors:** Saakshie Kumar, Indu B Dubey, Vridhi Chand Aggarwal, Rajesh K Soni

**Affiliations:** 1 Department of General Surgery, Vardhman Mahavir Medical College and Safdarjung Hospital, New Delhi, IND

**Keywords:** veress needle, port site hematoma, laparoscopic surgery, hasson's technique, pneumoperitoneum

## Abstract

Background

The creation of pneumoperitoneum is the first step in any laparoscopic surgery. There are various methods of creating pneumoperitoneum which can be divided into open or closed methods. The closed method involves the blind insertion of the Veress needle into the peritoneal cavity. The open technique involves making an incision and then dissecting the fascia to the peritoneal cavity to introduce the cannula under direct vision. This study was conducted to evaluate the safety and efficacy of open (Hasson’s) and closed (Veress) techniques of intraperitoneal access for the creation of pneumoperitoneum in laparoscopic surgery.

Material and methods

The study was conducted in the Department of General Surgery, Vardhman Mahavir Medical College and Safdarjung Hospital, New Delhi. This was a prospective observational study and a total of 100 patients of laparoscopic surgeries fulfilling inclusion criteria were included in the study - 50 patients in group A undergoing the open method of creating pneumoperitoneum and 50 patients in group B undergoing the closed method of creating pneumoperitoneum were evaluated for the study period of 18 months from October 2020 through June 2022.

Results

The mean time to create pneumoperitoneum was 5.3 ± 1.41 minutes in the open method and 6.21 ± 1.36 minutes in the closed method. The mean time for umbilical port closure in our study was 7.33 ± 1.66 in the open group and 8.86 ± 2.19 in the closed group. In our study, there was no vascular or visceral injury noted in either of the methods used for the creation of pneumoperitoneum. Post-operative complications were almost equal in both the groups.

Conclusions

Both open and closed methods of intraperitoneal access are safe and effective for the creation of pneumoperitoneum during abdominal laparoscopy. The open method of creating pneumoperitoneum in laparoscopic surgery is a quicker method for the creation of pneumoperitoneum as compared to the closed method of intraperitoneal access.

## Introduction

The word laparoscopy originated from the Greek words (Laparo - flank, Skopein - to examine). Laparoscopy is the art of examining the abdominal cavity and its contents. This is achieved by sufficiently distending the abdominal cavity (pneumoperitoneum) and visualizing the abdominal contents using an illuminated telescope. The major difference between laparoscopic surgery and conventional open surgery is the minimal access to the abdominal cavity, as the abdominal incision (and its associated complications) is replaced by very small incisions only sufficient to introduce a trocar of 5-10mm in diameter. By this minimal traumatic insult to the patient, if achieved safely and efficiently and there are no other complications, the patient’s postoperative recovery will be shorter with less pain and he will return to full activity and work in a shorter period of time. The main challenge facing the laparoscopic surgery is the primary abdominal access, as it is usually a blind procedure and associated with vascular and visceral injuries. It has been proved from studies that 50% of laparoscopic major complications occur before the commencement of the surgery.

Creating pneumoperitoneum is the first step in carrying out laparoscopic surgery for diagnostic and therapeutic purposes. The establishment of pneumoperitoneum requires the introduction of a sharp insufflating needle or trocar. Methods available for creating pneumoperitoneum and inserting the laparoscope at the beginning of the laparoscopic procedure can be divided into open or closed entry techniques [1]. The closed method is the traditional one, while the open method is a more recent one [2]. The closed technique involves the blind insertion of the Veress needle into the peritoneal cavity [3]. The open method involves making an incision and then dissecting the fascia to the peritoneal cavity to introduce the cannula under direct vision [4].

Both techniques are being used at different centers and a few studies have been published comparing their advantages and disadvantages, but no technique has been shown to be superior to the other [5]. The current study was conducted to evaluate the safety and efficacy of open (Hasson’s) and closed (Veress) techniques of intraperitoneal access for the creation of pneumoperitoneum in laparoscopic surgery in terms of average time taken for creation of pneumoperitoneum, complications related to the intraperitoneal access like vascular injury/bowel injury/omental injury, port site gas leakage, extra-peritoneal insufflations, average time taken for umbilical port closure and to assess post-operative complications like port site hematoma, subcutaneous emphysema, port site surgical site infections.

## Materials and methods

This prospective observational study was conducted in the Department of General Surgery, Vardhman Mahavir Medical College and Safdarjung Hospital, New Delhi from October 2020 through June 2022. The study of Moberg et al. [6] observed that the median and range values of time for access for residents in the Veress technique were 120 (49-600) seconds. Taking these values as a reference and assuming a difference of 100 seconds in time for access between open and closed techniques, the minimum required sample size with 80% power of study and 5% level of significance was 46 patients in each study group [6]. Patients were recruited till at least 50 patients were there in each group. A total of 100 patients of laparoscopic cholecystectomies of ASA (American Society of Anesthesiologists) grade 1, age >18 years and BMI <35 were included in the study, and patients with previous surgical procedures of the abdomen and associated comorbidities were excluded from the study. Patients were divided into two groups, 50 each of open and closed methods of creating pneumoperitoneum on the basis of the surgeon’s preference and expertise. One surgeon used the open method and the other used the closed method to create pneumoperitoneum. Each patient's details were kept in closed envelopes separately. Both the surgeons randomly fetched one envelope each and performed the surgery using their respective methods. Patients were recruited in the study till at least 50 cases belonged to each technique. In both the groups, time was noted to create pneumoperitoneum, and time for umbilical port closure was also noted. Any vascular or visceral injury was noted. Post-op complications such as port site hematoma, port site infection and port site skin pain were assessed. Surgical Site Infection (SSI) score was assessed on the basis of the Southampton Wound Scoring System and port site pain was assessed on the basis of the Visual Analogue Scale.

In Hasson’s technique, a small, 1.5-2 cm, horizontal or vertical incision was made either inferior or superior to the umbilicus. The incision was extended down through the subcutaneous tissues until the abdominal fascia can be grasped and elevated. The peritoneum was then incised under direct visualization. After confirmation of intra-peritoneal access and ensuring that there is no evidence of adhesions or injury to underlying organs, a 10-mm laparoscopic trocar was introduced into the abdomen. The anchoring stitches were then tightened onto the struts to further secure the trocar.

In the Veress technique, the Veress was checked prior to usage by retracting the sheath and ensuring that it springs back into place when released. In order to use the closed technique, a small incision was made either superior or inferior to the umbilicus with the patient in the supine position. The subcutaneous tissue was bluntly pushed aside using gauze, and the umbilicus was then grasped with a retractor and elevated. The needle was inserted either at 45° or 90° angle. A click was heard after moments of resistance when the needle passed through the peritoneum and the blunt tip retracted. After ensuring correct placement using a drop test and free mobility of the needle in the abdomen without any resistance Veress needle was connected to the CO2 insufflator and pneumoperitoneum was created up to 13 mmHg.

The data was collected and entered in a master chart and comparative analysis was made in terms of average time taken for the creation of pneumoperitoneum, average time taken for umbilical port closure, incidence of port site hematoma, incidence of visceral/vascular injury, incidence of port site infection, port site skin pain on day 1, day 10 and day 30.

## Results

The mean time to create pneumoperitoneum in the present study was 5.3 ± 1.41 minutes in the open method and 6.21 ± 1.36 minutes in the closed method as shown in Table 1 and the difference was highly statistically significant (p-value 0.0002).

**Table 1:** Time taken to create pneumoperitoneum (minutes) ‡ Independent t-test, † Chi-square test

Time taken to create pneumoperitoneum (minutes)	Open (n=50)	Closed (n=50)	Total	P-value
2 to 4 minutes	11 (22%)	2 (4%)	13 (13%)	0.005^†^
4.1 to 6 minutes	28 (56%)	25 (50%)	53 (53%)	
>6 minutes	11 (22%)	23 (46%)	34 (34%)	
Mean ± SD	5.13 ± 1.41	6.21 ± 1.36	5.67 ± 1.49	0.0002^‡^

Time taken for umbilical port closure in both the groups is shown in Table 2 and ranged from 4.8-11.6 minutes in the open group and 5.4-13.6 minutes in the closed group. The mean time for umbilical port closure in our study was 7.33 ± 1.66 in the open group and 8.86 ± 2.19 in the closed group as shown in Table 2 and the difference was highly statistically significant (p-value 0.0002).

**Table 2:** Time taken for umbilical port closure in both the groups ‡ Independent t-test, * Fisher's exact test

Time taken for umbilical port closure (minutes)	Open (n=50)	Closed (n=50)	Total	P-value
4 to 6 minutes	11 (22%)	6 (12%)	17 (17%)	0.005^*^
6.1 to 8 minutes	25 (50%)	13 (26%)	38 (38%)	
8.1 to 10 minutes	11 (22%)	19 (38%)	30 (30%)	
10.1 to 12 minutes	3 (6%)	6 (12%)	9 (9%)	
>12 minutes	0 (0%)	6 (12%)	6 (6%)	
Mean ± SD	7.33 ± 1.66	8.86 ± 2.19	8.09 ± 2.08	0.0002^‡^

Port site hematoma occurred in 2 (4%) patients of the open group and 1 (2%) patient of the closed group. There was no significant difference between the two groups in relation to port site hematoma. There was no vascular or visceral injury in the open or closed method of creating pneumoperitoneum. Two patients developed port site infection in each group which was successfully treated. The difference in port site pain was not statistically significant (p=0.613).

## Discussion

In the era of modern surgery, laparoscopic surgery has gained much popularity due to its advantages like minimal access approach, shorter hospital stay, early return to daily activity, minimal post-operative morbidity and good cosmesis [1]. Port placement and creation of pneumoperitoneum is the essential key step in laparoscopic surgery [7].

In the study by Chotai et al., the mean age of patients in the Veress needle group was 37.46 ± 12.9171 years and in the open method group, it was 39.80 ± 13.9477 years [8]. The mean age in our study is 35.32 ± 10.5.

There were 42 females in the open group and 40 females in the closed group. There were eight males in the open group and 10 males in the closed group. Female preponderance in our study might be because we have only taken cases of cholecystectomy.

The time taken to create pneumoperitoneum ranged from 2.9-8.8 minutes in the open method and 3.8-9.4 minutes in the closed method. In the study by Channa et al., the time to establish pneumoperitoneum was much less in the Hasson cannula technique (4.6 ± 1.1 minutes) as compared to the Veress needle technique (5.4 ± 0.7 minutes) [2]. In the study by Chotai et al., the time for the creation of pneumoperitoneum was 5.12 ± 2.5172 minutes in the closed method whereas it was 3.94 ± 2.774 minutes in the open method [8]. The mean time to create pneumoperitoneum in the present study was 5.3 ± 1.41 minutes in the open method and 6.21 ± 1.36 minutes in the closed method which is comparable with the above-mentioned studies.

The mean time taken for umbilical port closure was 7.33 ± 1.66 minutes in the open group and 8.86 ± 2.19 minutes in the closed group. Akbar et al. reported that the mean wound closure time was 9.88 minutes in group A (Veress needle) and 4.97 minutes in group B (Open method) [9]. In the study by Sangrasi et al., port wound closure time was also significantly less in the open group (7.41 ± 1.87 minutes) as compared to the closed group (10 ± 2.44 minutes) [10]. The mean time for umbilical port closure in our study was 7.33 ± 1.66 in the open group and 8.86 ± 2.19 in the closed group which is comparable with the above-mentioned studies.

Port site hematoma occurred in 2 (4%) patients of the open group and 1 (2%) patient of the closed group. In the study by Channa et al., port site hematoma occurred in 1 (1.7%) case in the open group and none in the closed group [2]. In the study by Jamil et al., port site hematoma occurred in 1 (0.23%) case in the Veress needle group and 3 (0.70%) cases in the Hasson’s cannula group [4]. There was no significant difference between both the groups in relation to port site hematoma.

There was no vascular or visceral injury in the open or closed method of creating pneumoperitoneum. Two patients developed port site infection of grade I Clavien-Dindo in each group which was successfully treated. In our study, there was no significant difference in the development of port site infection in both open and closed methods of creating pneumoperitoneum.

All patients of both the groups complained of port site pain on post-operative day 1 which was measured on the Visual Analogue Scale. In the study by Chotai et al., 100% of patients complained of port site pain [8].

The main limitation of this study was the number of patients. Complications of laparoscopic cholecystectomy have low incidence, thus safety profile of both the techniques could not be studied comprehensively. However, the sample suited the objectives of this study with regard to most of the variables. Another limitation is that this was a single-center study and like all single-center trials, the results cannot be widely generalized. In our study, cases of only laparoscopic cholecystectomies were studied, so the safety and efficacy of techniques used to create pneumoperitoneum on other laparoscopic surgeries still remain uncertain.

## Conclusions

Our study concluded that both open and closed methods of intraperitoneal access are safe and effective for the creation of pneumoperitoneum during abdominal laparoscopy. The open method of creating pneumoperitoneum in laparoscopic surgery is a quicker method for the creation of pneumoperitoneum as compared to the closed method of intraperitoneal access. Closure of the umbilical port requires less time as compared to the Veress technique of intraperitoneal access when sutures are preplaced at the time of open access. Intraoperative/postoperative complications like port site hematoma, vascular, visceral injury, port site infection or port site skin pain are negligible in expert hands using both the methods.
